# Large-Scale Structural Variation Detection in Subterranean Clover Subtypes Using Optical Mapping

**DOI:** 10.3389/fpls.2018.00971

**Published:** 2018-07-17

**Authors:** Yuxuan Yuan, Zbyněk Milec, Philipp E. Bayer, Jan Vrána, Jaroslav Doležel, David Edwards, William Erskine, Parwinder Kaur

**Affiliations:** ^1^School of Biological Sciences, The University of Western Australia, Perth, WA, Australia; ^2^Institute of Agriculture, The University of Western Australia, Perth, WA, Australia; ^3^Institute of Experimental Botany, Centre of the Region Haná for Biotechnological and Agricultural Research, Olomouc, Czechia; ^4^Centre for Plant Genetics and Breeding, School of Agriculture and Environment, The University of Western Australia, Perth, WA, Australia; ^5^Telethon Kids Institute, Perth, WA, Australia

**Keywords:** structural variation, optical mapping, Bionano, nucleotide validation, reference

## Abstract

We selected two genetically diverse subspecies of the *Trifolium* model species, subterranean clover *cvs*. Daliak and Yarloop. The structural variations (SVs) discovered by Bionano optical mapping (BOM) were validated using Illumina short reads. In the analysis, BOM identified 12 large-scale regions containing deletions and 19 regions containing insertions in Yarloop. The 12 large-scale regions contained 71 small deletions when validated by Illumina short reads. The results suggest that BOM could detect the total size of deletions and insertions, but it could not precisely report the location and actual quantity of SVs in the genome. Nucleotide-level validation is crucial to confirm and characterize SVs reported by optical mapping. The accuracy of SV detection by BOM is highly dependent on the quality of reference genomes and the density of selected nickases.

## Introduction

Subterranean clover is the key forage legume in Australia, producing valued feed for livestock on a sown area of more than 29 million hectares (Nichols et al., 2013). As with other legumes, symbiotic nitrogen fixation in subterranean clover contributes to soil improvement. Subterranean clover is diploid (2n = 2x = 16) with a genome size around 556 Mb/1C. Its inbreeding nature, annual habit, and well-assembled reference genome (*subterraneum*) have established it as a model for *Trifolium* (Nichols et al., 2013). Based on morphology, genetic, and cytogenetic data, subterranean clover is classified into three subspecies: *subterraneum*, *yanninicum*, and *brachycalycinum* (Katznelson and Morley, 1965a,b). The subspecies differ morphologically, enabling them to adapt to different soil environments, e.g., ssp. *subterraneum* and ssp. *yanninicum* are adapted to moderately acidic soils, with ssp. *subterraneum* found on well-drained soils and ssp. *yanninicum* adapted to water-enriched soils (Francis and Devitt, 1969). In contrast, ssp. *brachycalycinum* is adapted to dry and neutral-to-alkaline soils that contain cracks or stones facilitating burr development. In this study, we examined the sympatric subspecies *subterraneum* and *yanninicum* to check the performance of optical mapping in structural variation (SV) detection and validate the findings using short read sequencing.

Structural variations are genomic alterations in sequence size, copy number, orientation or chromosomal location between individuals (Feuk et al., 2006). They are important genetic features that enrich genetic diversity and lead to important phenotypes (Escaramis et al., 2015).

We selected the *Trifolium* model species, subterranean clover (*Trifolium subterraneum* L.), with a high-quality reference and high-resolution Bionano optical maps (BOM) for two genetically diverse subspecies. These BOM findings were then validated by high coverage Illumina short read data generated for the two subtypes.

## Materials and Methods

### Purification of Cell Nuclei

Suspensions of intact cell nuclei were prepared following Vrána et al. (2016). Approximately 20 g each of mature dry seeds of ssp. *subterraneum* cultivar Daliak and ssp. *yanninicum* cultivar Yarloop were germinated at 25°C on moist paper towels in a dark environment. When the roots reached 2–3 cm in length, they were excised about 1 cm from the root tip, fixed in (2% v/v) formaldehyde at 5°C for 20 min, and subsequently washed three times with Tris buffer (5 min each time). The root tips (∼40/sample) were excised and transferred to 1 ml IB buffer (Šimková et al., 2003), in which cell nuclei were isolated using a homogenizer at 13,000 rpm for 18 s. Large debris was removed by filtering through 50-μm nylon mesh, and the nuclei in suspension were stained with DAPI (2 μg ml^-1^).

### Preparation of High Molecular Weight (HMW) DNA

High molecular weight (HMW) DNA was prepared according to Šimková et al. (2003) with modifications. Four batches of 700,000 G1-phase nuclei each were sorted into 660 μl IB buffer in 1.5 ml polystyrene tubes using a FACSAria II SORP flow cytometer and sorter (BD Biosciences, San Jose, CA, United States). One 20 μL agarose miniplug was prepared from each batch of nuclei. The miniplugs were treated by proteinase K (Roche, Basel, Switzerland), washed in wash buffer (10 mM Tris, 50 mM EDTA, pH 8.0) four times, and subsequently five times in TE buffer (10 mM Tris, 1 mM EDTA, pH 8.0). After the plugs had been melted for 5 min at 70°C and solubilized with GELase (Epicentre, Madison, WI, United States) for 45 min, DNA was purified by drop dialysis against TE buffer (Merck Millipore, Billerica, MA, United States) for 90 min.

### Construction of Bionano Optical Map

The latest genome assembly of *T. subterraneum* (*cv.* Daliak) (Kaur et al., 2017) was used as a reference and digested *in silico* using Knickers (v1.5.5). Four available nickases (*Nt.BspQI:* GCTCTTC, *Nb.BbvCI*: CCTCAGC, *Nb.BsmI*: GAATGC, *Nb.BsrDI*: GCAATG) were used to check the frequency of enzyme restriction sites in the reference genome with *Nt.BspQI*, being the most appropriate enzyme to nick the HMW DNA with the expected frequency of 7.1 sites per 100 kb. In all Bionano experiments, *Nt.BspQI* was used. The DNA was labeled and stained following the manufacturer’s NLRS protocol as described in Kaur et al. (2017). Four runs on the Bionano Irys^®^ instrument (30 cycles/run) were carried for subspecices *yannicum* (*cv.* Yarloop) to achieve sufficient genome coverage (∼425 ×).

The dedicated Bionano IrysView (v2.5.1.29842), Bionano tools (v5122), Bionano scripts (v5134) and runBNG (Yuan et al., 2017b) were used to *de novo* assemble *cv.* Yarloop single molecule optical maps. Before *de novo* assembly, molecule quality was checked by running the Molecule Qlty Report (MQR) in Bionano IrysView using *cv.* Yarloop raw BOM data and the digested reference genomes. In the alignment parameter settings, the *p*-value (–T) was set to 1.81 × 10^-08^ and the number of iterations (–M) was set to 5. On receipt of the MQR, we followed the instruction of parameter settings from BioNano Genomics and adjusted the *de novo* assembly parameters from the default false positive density (–FP) 1.5 to 1.67, default negative rate (–FN) 0.15 to 0.09, default scalingSD (–*sd*) 0.0 to 0.25, default siteSD (–*sf*) 0.2 to 0.15, and default initial assembly *p*-value (–T) 1 × 10^-9^ to 1.81 × 10^-08^.

### Structural Variation Detection by BOM Validated Using Illumina Short Reads

After *de novo* assembly, runBNG was used for SV calling with default parameter settings (medium configration settings from Bionano Genomics). To check the accuracy of the SVs detected, we followed the pipeline from Shelton et al. (2015) using the default, strict and relaxed parameters in optical mapping and then performed SV calling. To further confirm the findings, we selected short paired-end reads for validation. The plants were grown in the field at Shenton Park, Western Australia (31°57′ S, 115°50′ E) and the genomic DNA was extracted from a single plant of *cv.* Yarloop and *cv.* Daliak - representatives of two subterranean clover subspecies. Truseq Illumina libraries were prepared with an insert size of approximately 550 b and the short paired-end reads were generated using Illumina HiSeq 2000 at coverage of 48 × in *cv.* Yarloop and 56 × in *cv.* Daliak [the same dataset used in Kaur et al. (2017)]. Reads from both cultivars were aligned to the latest nucleotide reference (*cv.* Daliak) respectively (Kaur et al., 2017) using Speedseq (v0.1.0) (Chiang et al., 2015). Results were visualized using the integrative genomics viewer (IGV) (v2.3.91) (Robinson et al., 2011). Nucleotide-level SV calling was performed using Lumpy (v0.2.11) (Layer et al., 2014) by analyzing the mapping result from Speedseq (v0.1.0) (Chiang et al., 2015). The settings of Speedseq reads mapping were the deafult. The program used from Lumpy was ‘lumpyexpress’. The nucleotide reference was the same one used in the Speedseq reads mapping. The short sequence reads of *cv.* Yarloop were the same as used in reads mapping. When large-scale regions containing SVs from BOM were identified, we checked the corresponding regions to see if these regions contain SVs from the results of Lumpy and the visualization by IGV.

## Results

### *De Novo* Assembly of *cv.* Yarloop Optical Map

A total of 1,083,671 single molecule maps (directly from the Irys platform without filtering) were generated with a total length of 235.5 Gb (∼48 × genome coverage), of which the molecule N50 was 212.7 kb, and the average label density was 7.5 per 100 kb (**Table 1**). After filtering maps < 150 kb, 958,136 single molecule maps remained with a total length of 212.7 Gb (∼385 × genome coverage), of which the molecule N50 was 218.6 kb, and the average label density was 8.3 per 100 kbp. Using the filtered single molecule maps, 375,975 single molecule maps were finally *de novo* assembled to generate 377 consensus maps. The total length of the generated consensus maps was 475 Mb (∼89% of the total length of the reference genome) with a map N50 of 1.8 Mb.

**Table 1:** Statistics of *cv*. Yarloop Bionano optical maps.

Subject	Raw Bionano data	Filtered Bionano data	Assembled Bionano data
Number of molecules	1,083,671	958,136	375,975
Number of consensus maps	N/A	N/A	377
Total length	235.5 Gb	212.7 Gb	475.2 Mb
N50^†^	212.7 kb	218.6 kb	1.8 Mb
Average of label density (/100 kb)	7.5	8.3	8.0
Coverage	425	385	0.89


*^†^In the set of molecules, N50 represents the length of the shortest molecule whose length is greater than half of the total sum of lengths of all molecules; it is the point of half of the mass of the distribution.*

### SVs Assessment With BOM Validated by Illumina Short Reads

The mapping rate between *cv.* Yarloop Bionano consensus maps and the *cv.* Daliak NGS reference is 7.4%. Bionano SV calling between *cv.* Yarloop Bionano molecule maps and the *cv.* Daliak reference genome identified 12 regions (tens of kb regions) containing deletions and 19 containing insertions in *cv.* Yarloop compared to *cv.* Daliak (Supplementary Figure S1). The average deletoin length was 6.2 kb (Supplementary Table S1) and in these regions, 9.7% of the sequences were assembly gaps (Ns). The average length of the insertions was 8.04 kb, and the percentage of unknown sequences in these regions was 3.6%. Compared to the average coverage of all *cv.* Yarloop Bionano consensus maps (95.64 ± 23.52), the average coverge of the deletion and insertion regions in BioNano consensus maps is 101.12 ± 6.75. When checking the SVs called from the pipeline presented by Shelton et al. (2015) the results are consistent with our findings. Speedseq/Lumpy SV calling detected 20,887 deletions, 115 inversions, and 1,331 duplications in *cv.* Yarloop. Among those 20,887 deletions there are 71 (on average 2,533 b) that supported the 12 regions (on average 6,196 b) implied by BOM in *cv.* Daliak (Supplementary Figure S2 and Supplementary Table S2). Lumpy did not detect any insertions.

## Discussion

Although whole genome sequencing has been widely used to detect SVs, it is challenging to characterize large-scale SVs in a genome, particular for large-scale insertions. Current sequencing technologies can produce sequencing reads from a hundred to tens of thousands base pairs (Yuan et al., 2017a). However, they are still not long enough to span some variant regions (Li et al., 2011). Optical mapping, as an alternative, has promised the accuracy in SV detection (Li et al., 2017).

In the *de novo* assembly of *cv.* Yarloop Bionano single molecule maps, the total length of the consensus maps accounted for ∼89% of the estimated *T. subterraneum* genome size contrary to our expectation of ∼100%. This incomplete assembly could be caused by the low-quality single molecule map filtering step or the single map fragmentation due to the close proximity of *Nt.BspQI* restriction sites leading to DNA double-strand breaks in some DNA regions (Hastie et al., 2013). If the fragmented Bionano maps are repeats, they may collapse due to sharing the same nicking site partten. If there is no restriction sites in the fragmented maps, in the *de novo* assembly, they could be excluded leading to a smaller consensus map size.

The mapping rate between *cv.* Yarloop Bionano optical maps to the Daliak NGS reference indicates that the genomes of *cv.* Yarloop and *cv*. Daliak are diverged and most of the genome cannot be aligned. This could be an explanation that only 12 deletions and 19 insertions were detected. When checking the avergae coverage of the detected SV regions and the average coverage of the Yarloop Bionano consenus maps, the results suggeste that our findings are reliable. To get rid of the bias from one paramter settings in the *de novo* assembly step, we followed the pipeline presented by Shelton et al. (2015). Our results have also been supported.

**FIGURE 1:**
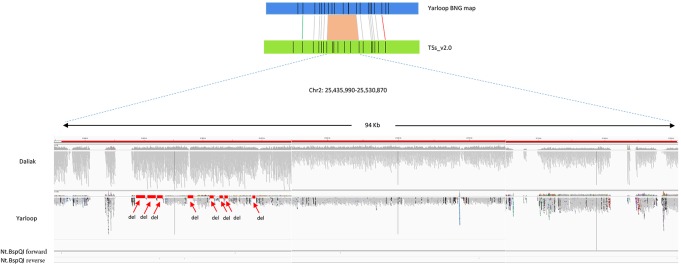
An example of deletions detected by Bionano optical mapping with nucleotide sequences validation. The region reported by Bionano contains deletion(s) in *cv*. Yarloop compared to the reference genome between location 25,435,990 b and 25,530,870 b in chromosome 2. TThe gray bars in this figure represents short reads aligned to the reference. Other color dots means different SNPs. Nt.BspQI forward represents sequence: GCTCTTC and Nt.BspQI reverse represents sequence: GAAGAGC. The size of the deletion reported by Bionano is 6.6 kb. From the nucleotide-level validation, eight small deletions (displayed as ‘del’) were visualized in the IGV with no sequence reads aligned to the reference genome. The total size of those eight small deletions is 6.1 kb. The eight small regions were supported in the Lumpy SV calling. The regions having zero coverage are the gaps in the reference.

In the Lumpy SV calling, we identified 71 small deletions in the 12 large-scale regions reported by BOM (**Figure 1**). While, the total length of the 71 deleted genomic regions reported by Lumpy was close to the total length reported by BOM (71.7 kb vs. 74.4 kb respectively), some length differences remained, probably due to the incorrect gap size or misassemblies in the reference genome, or also could be due to the incomplete SV calling in Lumpy. Interestingly, the gaps in the detected SV regions which were highly likely caused by collapse in the repetitive regions, were complemented by the Bionano super-scaffolding process for the generation of the advanced reference assembly (Kaur et al., 2017).

No insertions were reported by Lumpy in the SV calling, it is probable that those sequences being novel in *cv.* Yarloop compared to the reference assembly based on the *cv.* Daliak. When nucleotide level alignments were carried out using short sequence reads from the *cv.* Yarloop with the *cv.* Daliak, Yarloop reads from genomic regions not present in the reference assembly (either Yarloop-specific or unassembled in the reference) could not be mapped. As such, SV could not be called in these regions. Such novel sequences were grouped as unmapped sequences, earlier abandoned by Lumpy in SV calling. This issue has also been reported previously by (Xia et al., 2016) for most reference based SV calling methods, which cannot efficiently report large-scale insertions if there are many novel sequences in the examined individuals.

## Conclusion

Based on the physical location of nicking sites, optical mapping provides an attractive method to detect SVs. Single molecule maps produced by optical mapping are long enough to span most of the large and complex genome regions that traditional sequencing technologies are unable to achieve. However, optical mapping has some limitations in discovering the precise location and actual number of SVs owing to enzyme physical locations. NGS is useful to characterize SVs identified by optical mapping.

Although optical mapping provides the total size of SVs in a detected region, the total size of those SVs can be misreported due to the inaccurate gap size in the reference genome and/or absent enzyme restriction site information in the gap regions. To improve SV detection and characterization, a high-quality reference genome is crucial. In the absence of a high-quality reference genome, possible nucleotide-level validation of those identified SV regions is recommended to assess the accuracy of SV calling in optical mapping.

## Availability of Data

All raw nucleotide data and Bionano data are under BioProject PRJNA404013.

## Author Contributions

PK, DE, PB, and YY conceived and designed the research. ZM, JV, and JD performed the Bionano Irys^®^ System genome mapping experiments. YY performed the bioinformatics analysis, prepared the figures and wrote the manuscript with contributions from PK, PB, ZM, WE, DE, JD, and JV. All authors read and approved this manuscript.

## Conflict of Interest Statement

The authors declare that the research was conducted in the absence of any commercial or financial relationships that could be construed as a potential conflict of interest.

## Abbreviations

BOM: Bionano optical mapping
DAPI: 4′,6-diamidino-2-phenylindole
DNA: deoxyribonucleic acid
MQR: molecule quality report
N/A: not available
NGS: next generation sequencing
SNP: single-nucleotide polymorphism
SV: structural variation
