# Supplementary material for: Large-Scale Structural Variation Detection in Subterranean Clover Subtypes Using Optical Mapping
Source: Front Plant Sci. 2018 Jul 17;9:971. doi: 10.3389/fpls.2018.00971 (PMC6056659; doi:10.3389/fpls.2018.00971)
Supplement: Supplementary file 1 [file Data_Sheet_1.DOCX]

Supplementary Material

**Large-scale structural variation detection in subterranean clover subtypes using optical mapping validated at nucleotide level**

**Yuxuan Yuan, Zbyněk Milec, Philipp E. Bayer, Jan Vrána, Jaroslav Doležel, David Edwards, William Erskine and Parwinder Kaur**

*** Correspondence:** Parwinder Kaur: parwinder.kaur@uwa.edu.au

# Supplementary Figures and Tables

## Supplementary Figures


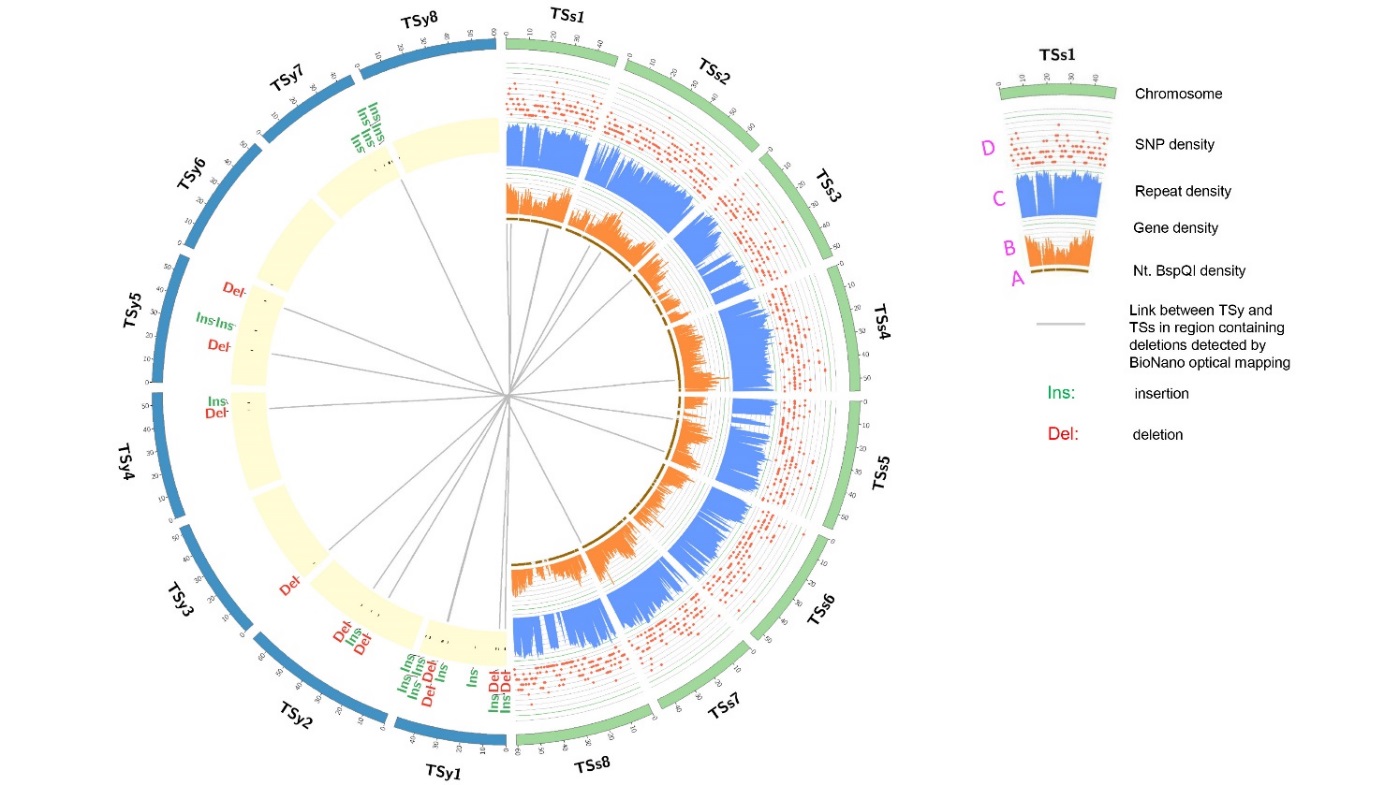


**Supplementary Figure 1.** Graphical view of the structural variations detected by BioNano in two subterranean clover subspecies: *subterraneum* (TSs) and *yanninicum* (TSy). A) Nt. BspQI density; B) Gene density; C) Repeat density D) SNP densities.The grey lines represent deletion regions linked between *cv.* Yarloop and *cv.* Daliak in corresponding chromosomes.


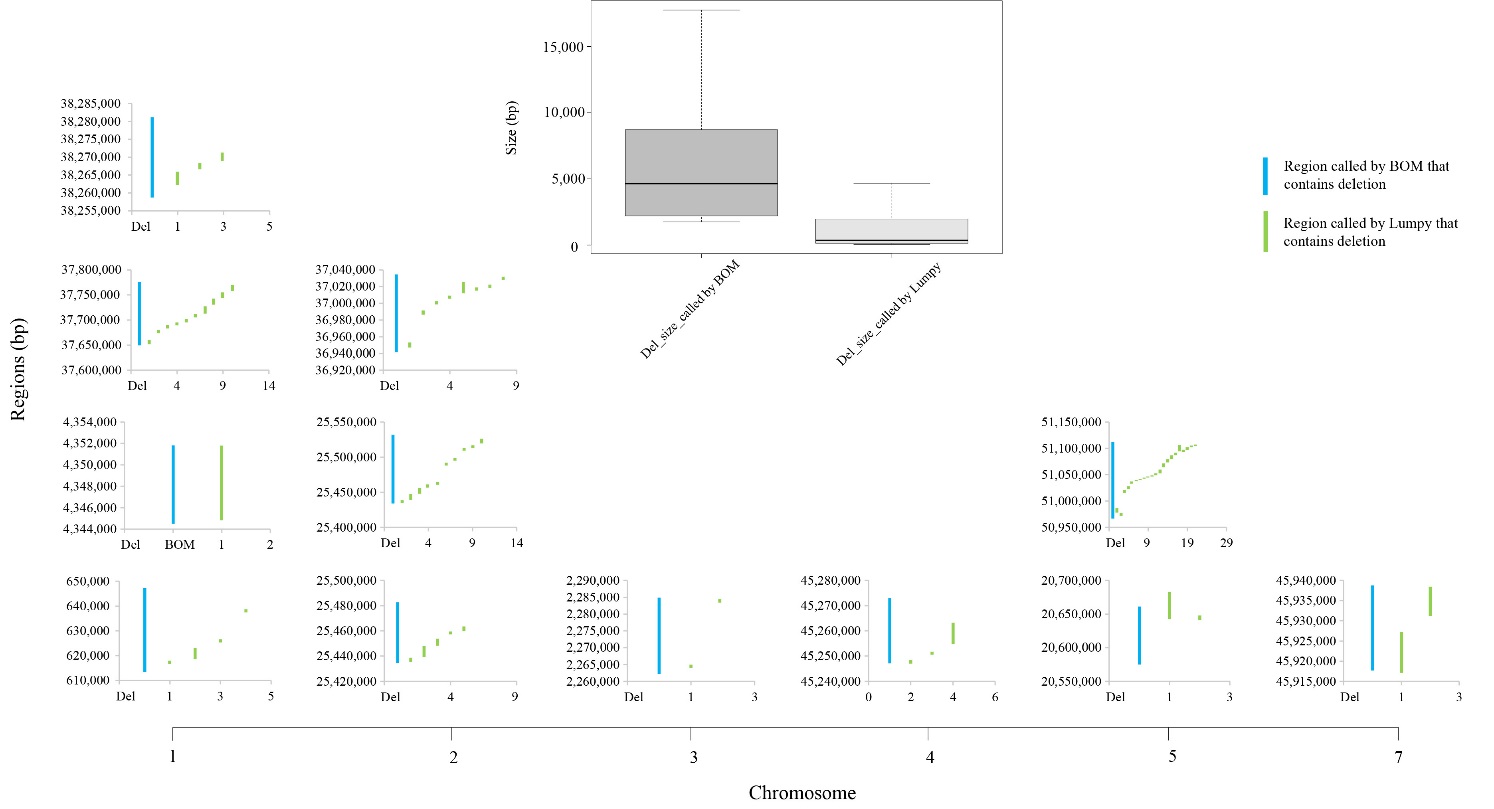


**Supplementary Figure 2.** Deletion regions detected by BioNano optical mapping and validated by next-generation sequencing. BioNano optical mapping (BOM) detected 12 large-scale regions containing deletions in 6 chromosomes with 71 small deletions supported by Illumina short read SV calling. In this figure the blue bars represent the regions detected by BOM that contain deletions. The green bars represent the deletions detected by Lumpy. The y-axes show the regions in corresponding chromosome that deletions occur. The sub-x-axes show the number of deletions detected by Lumpy supporting the BOM SV calling. The boxplots display the range of deletion size that detected by BOM and Lumpy.

## Supplementary Tables

**Supplementary Table S1.** Regions detected by BioNano optical mapping with deletions in *cv.* Yarloop compared to *cv.* Daliak

| Chr | Start | End | Deletion_size (bp) | Confidence_score |
| --- | --- | --- | --- | --- |
| 1 | 613810 | 647373 | 3234 | 0.95 |
| 1 | 4344632 | 4351753 | 1968 | 0.68 |
| 1 | 37655131 | 37766329 | 17749 | 0.95 |
| 1 | 38258051 | 38279873 | 2296 | 0.95 |
| 2 | 25435990 | 25481860 | 4770 | 0.96 |
| 2 | 25435990 | 25530870 | 6608 | 0.92 |
| 2 | 36943765 | 37033667 | 1743 | 0.71 |
| 3 | 2262447 | 2284706 | 4490 | 0.96 |
| 4 | 45247639 | 45272424 | 7775 | 0.92 |
| 5 | 20577425 | 20659779 | 11983 | 0.88 |
| 5 | 50970142 | 51111563 | 9650 | 0.92 |
| 7 | 45918098 | 45938652 | 2095 | 0.95 |

**Supplementary Table S2.** Deletions called by Illumina short reads in the regions detected by BioNano that contain deletions.

| Chr | Start | End | Deletion_size (bp) |
| --- | --- | --- | --- |
| 1 | 618523 | 618653 | 131 |
| 1 | 619610 | 622826 | 3217 |
| 1 | 626111 | 626174 | 64 |
| 1 | 638189 | 638253 | 65 |
| 1 | 4345004 | 4346687 | 1684 |
| 1 | 37656657 | 37659942 | 3286 |
| 1 | 37688543 | 37688641 | 99 |
| 1 | 37690277 | 37690544 | 268 |
| 1 | 37693910 | 37695133 | 1224 |
| 1 | 37695706 | 37701447 | 5742 |
| 1 | 37713376 | 37715109 | 1734 |
| 1 | 37715889 | 37721305 | 5417 |
| 1 | 37740630 | 37742606 | 1977 |
| 1 | 37760128 | 37760284 | 157 |
| 1 | 37760493 | 37760756 | 264 |
| 1 | 38263223 | 38265193 | 1971 |
| 1 | 38265309 | 38265371 | 63 |
| 1 | 38266765 | 38267882 | 1118 |
| 2 | 25436756 | 25437884 | 1129 |
| 2 | 25440601 | 25447857 | 7257 |
| 2 | 25448557 | 25453225 | 4669 |
| 2 | 25458667 | 25458717 | 51 |
| 2 | 25461232 | 25463269 | 2038 |
| 2 | 25436756 | 25437884 | 1129 |
| 2 | 25440601 | 25447857 | 7257 |
| 2 | 25448557 | 25453225 | 4669 |
| 2 | 25458667 | 25458717 | 51 |
| 2 | 25461232 | 25463269 | 2038 |
| 2 | 25490634 | 25490689 | 56 |
| 2 | 25498182 | 25498231 | 50 |
| 2 | 25512704 | 25512817 | 114 |
| 2 | 25514643 | 25514711 | 69 |
| 2 | 25520877 | 25524929 | 4053 |
| 2 | 36949771 | 36952852 | 3082 |
| 2 | 36989059 | 36990179 | 1121 |
| 2 | 37011397 | 37011576 | 180 |
| 2 | 37013423 | 37013551 | 129 |
| 2 | 37013680 | 37024736 | 11057 |
| 2 | 37017492 | 37017721 | 230 |
| 2 | 37019871 | 37021199 | 1329 |
| 2 | 37030176 | 37030580 | 405 |
| 3 | 2264705 | 2264824 | 120 |
| 3 | 2284037 | 2284288 | 252 |
| 4 | 45247716 | 45247768 | 53 |
| 4 | 45251307 | 45251482 | 176 |
| 4 | 45255357 | 45263088 | 7732 |
| 5 | 20644070 | 20683208 | 39139 |
| 5 | 20644110 | 20647241 | 3132 |
| 5 | 50980767 | 50984055 | 3289 |
| 5 | 50980861 | 50981202 | 342 |
| 5 | 51019581 | 51019663 | 83 |
| 5 | 51025991 | 51026344 | 354 |
| 5 | 51041165 | 51041404 | 240 |
| 5 | 51043826 | 51043889 | 64 |
| 5 | 51046599 | 51046793 | 195 |
| 5 | 51047185 | 51047523 | 339 |
| 5 | 51047782 | 51048283 | 502 |
| 5 | 51048889 | 51049037 | 149 |
| 5 | 51052743 | 51054078 | 1336 |
| 5 | 51054596 | 51057844 | 3249 |
| 5 | 51068509 | 51071320 | 2812 |
| 5 | 51077378 | 51077637 | 260 |
| 5 | 51084536 | 51088248 | 3713 |
| 5 | 51089998 | 51091336 | 1339 |
| 5 | 51097179 | 51105458 | 8280 |
| 5 | 51097640 | 51098564 | 925 |
| 5 | 51098800 | 51102232 | 3433 |
| 5 | 51103216 | 51103291 | 76 |
| 5 | 51103592 | 51104902 | 1311 |
| 7 | 45917490 | 45927220 | 9731 |
| 7 | 45931674 | 45938292 | 6619 |
